# A cancer therapeutic nanoparticle vaccine targeting HAAH improves 3-week survival from 12.5% to 100% in a mouse model of metastatic breast cancer

**DOI:** 10.1186/2051-1426-2-S3-P52

**Published:** 2014-11-06

**Authors:** Steven Fuller, Michael S Lebowitz, Solomon Stewart, Hossein A Ghanbari

**Affiliations:** 1 Panacea Pharmaceuticals, Inc., Gaithersburg, MD, USA

## 

Human aspartyl (asparaginyl) β-hydroxylase (HAAH) is over-expressed on the surface of cancer cells. It is an embryonic protein that is demonstrated to be responsible for cancer etiology: cell proliferation, motility and invasiveness. When normal cells are transfected to over-express HAAH they behave as transformed and when HAAH is neutralized or its expression is inhibited, cancer cells regain a normal phenotype. Anti-HAAH antibodies inhibit cancer cell growth, motility and invasiveness *in vitro*, and inhibit tumor growth *in vivo*. We have developed a novel anticancer nanoparticle vaccine in which the N-terminal or C-terminal thirds of HAAH are expressed on λ-phage (200-300 copies). These anticancer vaccines are immunogenic and produce high-titer anti-HAAH polyclonal antibodies in mice. The HAAH gene is highly conserved between mice and humans. In several experiments these vaccines have demonstrated strong tumor growth inhibition in animals. We have previously presented these findings in three posters at SITC 2013. More recently, we have tried to determine the efficacy of these vaccines in a model of metastatic breast cancer. After preliminary experiments which showed vaccine-induced inhibition of metastasis to the lung, we proceeded with the following experiment. Female mice were separated into three groups (n = 8). Groups 2 and 3 were immunized with 10^10 ^phage particles displaying the N-terminal or C-terminal third of human HAAH, respectively. Group 1 served as the control group and received the parent λ-phage, not displaying any added protein. Immunizations were performed on days 1, 8 and 22. Mice were injected with 2x10^4 ^breast cancer cells (4T1, mouse, triple-negative breast cancer line) subcutaneously into the mammary fat pad on day 15. To our surprise, some control animals died as early as day 30 of the experiment, only 15 days after tumor cell challenge. By the end of the third week post cancer cell transplantation (day 36), only 1 of 8 animals in the control group survived (12.5%) while 5 of 8 (67.5%) and 8 of 8 (100%) remained alive in groups 2 and 3, respectively. No further deaths occurred out to day 43, after which the experiment was terminated. The greater efficacy of the C-terminal vaccine is likely related to the fact that it comprises the catalytic domain of HAAH. This unintended "survival" experiment indicates that this unique nanoparticle formulation overcomes some of the hurdles to cancer vaccine development.

